# The IDI1/SREBP2 axis drives intrahepatic cholestasis and is a treatment target of San-Huang-Cai-Zhu formula identified by sequencing and experiments

**DOI:** 10.3389/fphar.2023.1093934

**Published:** 2023-02-08

**Authors:** Junbin Yan, Yunmeng Nie, Zheng Chen, Jiaming Yao, Shuo Zhang, Zhiyun Chen

**Affiliations:** ^1^ The First Affiliated Hospital of Zhejiang Chinese Medical University, Zhejiang Provincial Hospital of Chinese Medicine, Hangzhou, China; ^2^ The Second Affiliated Hospital of Zhejiang Chinese Medical University, The Xin Hua Hospital of Zhejiang Province, Hangzhou, China; ^3^ School of Basic Medical Sciences, Zhejiang Chinese Medical University, Hangzhou, China; ^4^ Key Laboratory of Integrative Chinese and Western Medicine for the Diagnosis and Treatment of Circulatory Diseases of Zhejiang Province, Hangzhou, China; ^5^ Hangzhou Hospital of Traditional Chinese Medicine, Hangzhou, China

**Keywords:** intrahepatic cholestasis, traditional Chinese medicine, San-Huang-Chai-Zhu formula, cholesterol metabolism, IDI1, SREBP2

## Abstract

San-Huang-Chai-Zhu formula (SHCZF), originates from Da-Huang-Xiao-Shi decoction (DHXSD) for the treatment of jaundice as recorded in the Chinese traditional Chinese medicine book *Jin Gui Yao Lue*. In the clinic, SHCZF has been used to treat cholestasis-related liver disease by improving intrahepatic cholestasis, but the treatment mechanism has not been elucidated. In this study, 24 Sprague-Dawley (SD) rats were randomly assigned to the normal, acute intrahepatic cholestasis (AIC), SHCZF, and ursodeoxycholic acid (UDCA) groups. In addition, 36 SD rats were divided into dynamic groups, namely, normal 24 h, AIC 24 h, normal 48 h, AIC 48 h, normal 72 h, and AIC 72 h groups. Alpha-naphthylisothiocyanate (ANIT) was used to induce an AIC rat model. Serum biochemical indices and hepatic pathology were detected. Part of the hepatic tissues was used for sequencing, and others were used for subsequent experiments. Sequencing data combined with bioinformatics analysis were used to screen target genes and identify the mechanisms of SHCZF in treating AIC rats. Quantitative real-time PCR (qRT-PCR) and Western blotting (WB) were used to detect the RNA/Protein expression levels of screened genes. Rats in the dynamic group were used to determine the sequence of cholestasis and liver injury. High-performance liquid chromatography (HPLC) was used to determine the representative bioingredients of SHCZF. Sequencing and bioinformatics analysis suggested that IDI1 and SREBP2 are hub target genes of SHCZF to ameliorate ANTI-induced intrahepatic cholestasis in rats. The treatment mechanism is associated with the regulation of lipoprotein receptor (LDLr) to reduce cholesterol intake and 3-Hydroxy-3-Methylglutaryl-CoA reductase (HMGCR), and 3-Hydroxy-3-Methylglutaryl-CoA synthase 1 (HMGCS1) to decrease cholesterol synthesis. Animal experiments showed that SHCZF significantly reduced the expression levels of the above genes and proinflammatory cytokine lipocalin 2 (LCN2), inflammatory cytokines interleukin 1 beta (IL-1β) and tumor necrosis factor alpha (TNF-α), thereby improving intrahepatic cholestasis and inflammation and liver injury.

## Graphical Abstract

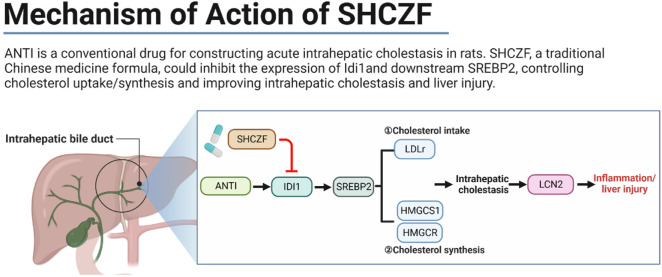

## Highlights

- Elevated expression of IDI1, and SREBP2 will cause intrahepatic cholestasis in rats.
- Excessive intrahepatic cholestasis may activate the expression of the pro-inflammatory gene LCN2, causing hepatic inflammation and injury.
- The effect of San-Huang-Cai-Zhu formula (SHCZF) in improving intrahepatic cholestasis is similar to that of Ursodeoxycholic Acid (UDCA). The effect of SHCZF in improving liver injury is better than UDCA.
- The regulatory pathways of SHCZF include IDI1/SREBP2 and its downstream LDLr (reduces free cholesterol uptake) and HMGCR/HMGCS1 (reduces cholesterol synthesis in the liver).

## 1 Introduction

Intrahepatic cholestasis (IHC) is a pathological process caused by hepatic-disordered bile synthesis/absorption (Meier-Abt, 1990). Clinically, the prevalence of IHC is high. 35% of patients diagnosed with chronic liver disease have concurrent IHC (Bortolini et al., 1992). Patients with cholestatic liver diseases (CLDs), intrahepatic cholestasis of pregnancy (ICP), neonatal cholestasis, or progressive familial intrahepatic cholestasis (PFIC) have a high probability of IHC (Balistreri, 1985; Gossard and Talwalkar, 2014; Williamson and Geenes, 2014; Hassan and Hertel, 2022). In addition, the diagnosis of cholestasis is difficult. In the early stages, patients with IHC present normal or slightly elevated serum alkaline phosphatase (ALP) and gamma-glutamyl transaminase (GGT). A series of clinical manifestations, such as jaundice, will only gradually emerge in the progressive stage (Kuntz and Kuntz, 2008). Prolonged IHC will induce further fibrosis and cirrhosis in CLD patients, eventually causing liver failure (Guo et al., 2020; Ginès et al., 2021).

Because IHC is classified as jaundice in Chinese medicine, Da-Huang-Xiao-Shi decoction (DHXSD), the traditional formula for jaundice treatment, which originated from Jin Gui Yao Lue, is usually used to treat IHC in clinics (He et al., 1990; Chen et al., 2018). DHXSD is composed of *Mirabilitum* (Mang xiao), *Phellodendron chinense Schneid.* (Huang bai), *Rheum officinale Baill.* (Da Huang) and *Gardenia jasminoides Ellis.* (Zhi Zi). Studies have shown that DHXSD effectively improves cholestasis in mice and rats (Zhu and Feng, 2019; Xue et al., 2021). We removed *Mirabilitum* (Mang xiao) in the clinical application instead of adding *Atractylodes macrocephala Koidz.* (Bai Zhu) and *Bupleurum chinensis DC.* (Chai Hu), which dredge the liver and strengthen the spleen. Thus, San-Huang-Chai-Zhu formula (SHCZF), originating from DHXSD, was formed.

We have confirmed that SHCZF can ameliorate IHC in rats by regulating the expression of CYP4A1, HACL1, and F11R (Yao et al., 2021) and dose-dependently improve IHC-induced liver injury by inhibiting SIRT1/PGC-1α and mitochondrial oxidative stress (Liu et al., 2022b). Through network pharmacology and molecular docking, we also identified possible bioactive components in SHCZF, such as chrysin and rhodopsin, that can target AKT1 and TP53 for the treatment of AIC in rats (Liu et al., 2022a). To provide more convincing data supporting the clinical promotion of SHCZF, we screened and validated the mechanisms of SHCZF in treating AIC from a more comprehensive perspective through sequencing and bioinformatics analysis.

## 2 Materials and animals

### 2.1 Preparation of SHCZF

SHCZF is composed of five traditional Chinese herbs: *Rheum officinale Baill*. (Da Huang), *Phellodendron chinense Schneid.* (Huang Bai), *Gardenia jasminoides Ellis.* (Zhi Zi), *Bupleurum chinensis DC.* (Chai Hu), and *Atractylodes macrocephala Koidz.* (Bai Zhu) with the ratio of 4 : 4 : 3 : 3 : 4. SHCZF was prepared by the pharmaceutical center of The First Affiliated Hospital of Zhejiang Chinese Medical University. The traditional decoction method (three-decoction) was used to extract as many bioactive ingredients from herbs as possible (Wang and Liu, 1997; Lin et al., 2011). The process is as follows: 1) add ten times the volume of water to the herbs, steep for 30 min, boil and decoct for 1.5 h; 2) add eight times the volume of water to the dregs, boil and decoct for 1 h; 3) add six times the volume of water to the dregs, boil and decoct for 0.5 h. The liquids obtained from the three decoctions were combined and concentrated into SHCZF extract (2 g·ml^−1^).

### 2.2 Bioactive ingredients analysis of SHCZF

High-Performance Liquid Chromatography with Tandem Mass Spectrometry (HPLC-MS/MS), used to analyze the bioactive ingredients of SHCZF, was performed by Qingdao Sci-tech Innovation Quality Testing Co., Ltd. liquid Chromatograph Mass Spectrometer.

100 mg SHCZF extract was diluted in 1 mL 70% methanol. The automatic grinder was used for crushing the above samples, followed by low-temperature sonication (40 KHZ) for 10 min. The mixture was centrifuged for 10 min (4°C, 12,000 rmp) to acquire the supernatant. Agilent Zorbax Eclipse C18 column (1.8 μm, 2.1 × 100 mm, USA) was used for chromatographic separation. The mobile phase consisted of A (0.1% formic acid aqueous solution) and B (pure acetonitrile). The column temperature was 30°C. The injection volume was 2 μL. The liquid chromatography (LC) mobile phase conditions are shown in Table 1.

**TABLE 1:** Liquid chromatography mobile phase conditions.

Time (min)	Flow rate (μL/min)	Gradient	B (%)	A (%)
0–2	300	/	5	95
2–6	300	Linear gradient	30	70
6–7	300	/	30	70
7–12	300	Linear gradient	78	22
12–14	300	/	78	22
14–17	300	Linear gradient	95	5
17–20	300	/	95	5
20–21	300	Linear gradient	5	95
21–25	300	/	5	95

Mass spectrometry (MS) monitoring was operated in positive and negative ion modes. The conditions of the different modes can be found in our previous studies (Hong et al., 2020). Refer to Thermo mzCloud and Thermo mzValut databases for identifying SHCZF bioactive ingredients.

### 2.3 Reagents of experiments

The reagents used in the experiments are as follows. Hepatic cholesterol test kit (E1015) was purchased from APPLYGEN. Protein Extraction Kit (KGP2100), 2xSDS-PAGE protein loading buffer (KGP1012) were purchased from KeyGEN Biotech. Bicinchoninic acid (BCA) protein assay kit (A20312), Goat Anti-Mouse IgG (H + L) HRP conjugated (GAM007) and Goat Anti-Rabbit IgG (H + L) HRP conjugated (GAR007) were purchased from MULTI SCIENCES. TGX Stain-Free™ FastCast ™ Acrylamide kit 7.5% (#1610181), TGX Stain-Free™ FastCast ™ Acrylamide kit 12% (#1610185), and Trans-Blot Turbo 5x Transfer Buffer (#10026938) were purchased from BIO-RAD. Polyvinylidene difluoride (PVDF) membranes (IPVH00010) were purchased fromMillipore. Anti-GAPDH monoclonal antibody (YM3029), IDI1 rabbit Pab (YT7476), SREBP2 Polyclonal antibody (YN0037), and LCN2 rabbit pAb (YT7924) were purchased from Immunoway. Tissue RNA purification kit plus (RN002plus) was purchased from ES Science. PrimeScript^™^ RT reagent Kit with gDNA Eraser (RR047A) and TB Green^®^ Premix Ex Taq^™^ II (RR820A) were purchased from Takara.

### 2.4 Establishment of animal model

**FIGURE 1:**
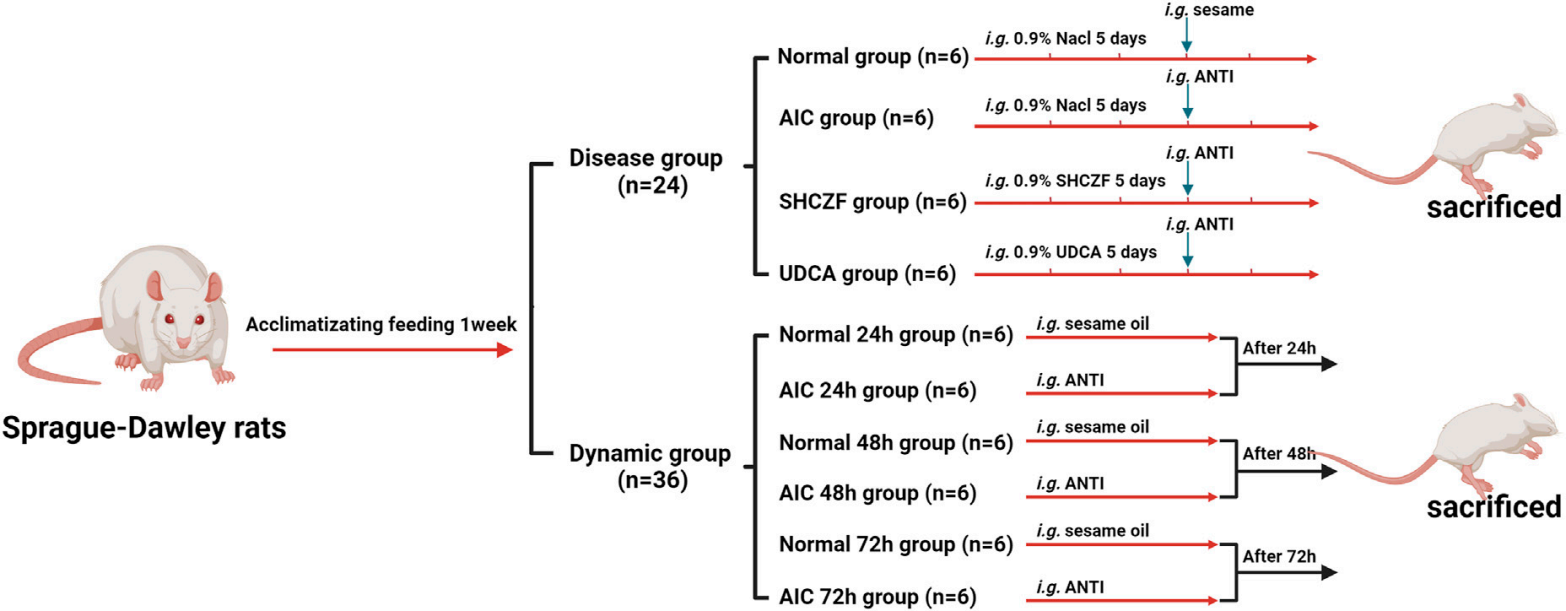
The flow of animal grouping and modeling.

A total of 60 male Sprague-Dawley (SD) rats (SPF grade, 8 weeks, 140 ± 10 g) were provided by Shanghai SIPPR-Bk Laboratory Animal Co., Ltd. (SCXK (Hu) 2013-0016). All rats were housed in an environment with a temperature of 22°C ± 2°C and humidity of 60 ± 5%. After 1 week of acclimatizing feeding, rats were randomly divided into disease (n = 24) and dynamic groups (n = 36). After receiving the appropriate interventions rats were intraperitoneally injected with 50 mg·kg^−1^ pentobarbital sodium and then sacrificed (Figure 1). Hepatic tissues and serum were collected for subsequent analysis. All animal research was conducted following Association for Assessment and Accreditation of Laboratory Animal Care (AAAALAC) and Institutional Animal Care and Use Committee (IACUC) guidelines and approved by the Animal Experimentation Ethics Committee of Zhejiang Chinese medical university (ZSLL-2015-195). The modeling conditions used in this study were long-used by the research group with literature support (Chisholm and Dolphin, 1996; Yao et al., 2021), resulting in low mortality (0%) and a high success rate of modeling (100%).

#### 2.4.1 Disease model and drugs intervention

The rats in the disease group were randomly divided into the normal, AIC, SHCZF, and UDCA groups (6 rats in each group). Rats in AIC groups were given 4% alpha-naphthylisothiocyanate (ANIT) (J&K Scientific, Lot: 249447) 100 mg·kg^−1^ (the dosage of ANTI referred to the literature and our previous studies) (Chisholm and Dolphin, 1996; Yao et al., 2021). Rats in the SHCZF and UDCA groups were gavaged 20 g·kg^−1^ of SHCZF or 90 mg·kg^−1^ of UDCA, respectively. The doses of the above therapeutic drugs are obtained by equivalence conversion concerning the optimal clinical using amounts.

#### 2.4.2 Dynamic model

The rats in the dynamic group were divided into normal 24 h, AIC 24 h, normal 48 h, AIC 48 h, normal 72 h, and AIC 72 h groups (6 rats in each group). Rats were respectively executed after injecting ANTI (100 mg·kg^−1^) 24 h, 48 h, and 72 h.

## 3 Sequencing and bioinformatics analysis

Further, we used hepatic tissues for sequencing to determine the treating mechanism and target genes of SHCZF. Based on whole-genome sequencing and bioinformatics analysis, we combined the existing literature to screen the potential mechanism and target genes of SHCZF in treating AIC rats.

### 3.1 Sequencing

Sequencing was performed by Shanghai Yuanzi Biotechnology Co., Ltd., of China. The analysis process was as follows: total hepatic RNA was extracted with TRIzol reagent (Invitrogen, USA). Total RNA was analyzed for purity and concentration using an Agilent 2100 Bioanalyzer and RNA 6000 Nano LabChip Kit (Agilent, USA). Quality RNA was amplified to construct libraries, which were subsequently sequenced by Illumina high-throughput sequencing analysis.

### 3.2 Bioinformatics analysis

#### 3.2.1 Screening of target genes

The genes with zero expression were deleted, while log2 (exp+1) transformation was performed. Boxplot, partial least squares discrimination analysis (PLS-DA), and dendrogram (R package ropls and ggplot2 were selected (Thévenot et al., 2015)) were used to determine whether the genes' expression data needed to be normalized and whether there was an expression convergence between groups and differences outside the groups.

Rats were screened for differential expressed genes (DEGs) before and after modeling (Normal vs. AIC) and treatment (AIC vs. SHCZF), respectively, using the ebayes analysis of the R package limma (Ritchie et al., 2015). |logFC|>1.5, *p*-value < 0.05 were used as the screening condition. Genes with opposite expression changes in Normal vs. AIC and AIC vs. SHCZF were intersected to screen for genes whose expression significantly changed in the progression of Normal to AIC and improved after SHCZF intervention. These intersected genes were considered target genes of SHCZF to improve AIC. The expression changes of the above genes in different groups of rats were further analyzed.

#### 3.2.2 Screening of treatment mechanisms

The genes with expression changes before and after modeling (Normal vs. AIC) and treatment (AIC vs. SHCZF) (|logFC|>0, *p*-value < 0.05) were analyzed by Gene Set Enrichment Analysis (GSEA). C2 gene sets of the MSigDB database ( *https://www.gsea-msigdb.org/gsea/msigdb/human/collections.jsp#C2* ) were used as background data sets to infer which genes and pathways are most closely related to the disease and SHCZF intervention. Gene sets in the collection are curated from various sources, including online pathway databases and literature. The top three pathways with the lowest *p*-value will be further analyzed.

## 4 Experimental validation

### 4.1 Biochemical indices

After the rats were anesthetized, blood was taken from abdominal veins. Centrifugation was used to separate serum. ALT, AST, ALP, TPA, and TBIL levels were detected with an automatic biochemical analyzer. ALT and AST were used to reflect the degree of hepatic injury in rats. ALP, TPA, and TBIL values were used to reflect the intrahepatic cholestasis in rats.

### 4.2 Liver histopathological analysis

#### 4.2.1 Hematoxylin and eosin (HE) staining

Rat hepatic tissues were fixed with 10% formaldehyde for 48 h and dehydrated with alcohol. Then, hepatic tissues were embedded in paraffin and cut into sections with a thickness of 4 μM (Leica, Germany). After dewaxing and rehydration, the sections were stained with hematoxylin for 5 min and eosin for 2 min. Finally, the sections were observed under an optical microscope (Olympus, Japan).

#### 4.2.2 Immunohistochemistry to detect CK19

Paraffin-embedded sections of rat liver tissue were first dewaxed and hydrated. Then, antigen repair and blocking were performed. The sections were incubated separately with primary antibody (overnight at 4°C) and secondary antibody (1 h at room temperature). DAB was used as a staining agent. Hematoxylin was used for restaining. At last, the staining of sections was observed under an optical microscope (Olympus, Japan).

### 4.3 Hepatic cholesterol content analysis

Before lysis, PBS was used to wash hepatic tissues twice to remove residual blood. Adding 10 µL lysate per 1 mg of tissue. The supernatant was heated at 70°C for 10 min, and then centrifuged at room temperature of 2,000 g for 5 min. The supernatant was used for TC detection. The BCA method was used to determine the protein concentration in the above tissues. The ratio of cholesterol and protein concentrations was seen as the cholesterol content in rat liver tissue.

### 4.4 Western blot analysis

A total protein extraction kit was used to extract proteins from hepatic tissues. Protein concentrations were determined using the BCA method and uniformly diluted to 8 ng/μL. SDS loading buffer (×2) was used to configure the protein for electrophoresis. The loading protein (40 ng) was added to SDS-PAGE gels (7.5% or 12.5%). Electrophoresis was performed for 50 min at a consistent voltage (180 V). Then, the proteins were transferred to PVDF membranes using a semi-dry transfer machine (Bio-Rad, USA). PVDF membranes were incubated with the corresponding primary antibodies overnight (4°C). Primary antibodies were used at the described concentrations: LCN2 (1:800), SREBP2 (1:1000), IDI1 (1:1500), and GAPDH (1:3000). The next day, the PVDF membranes were incubated with secondary antibody (1:5000) for 1 h at room temperature. An Odyssey Infrared Imaging System was used to analyze images and corresponding gray values. Subsequently, Spearman correlation analysis was used to confirm correlations between the relative protein expression of IDI1, SREBP2 and AIC related biochemical indicators (ALP, AST, ALT) to further judge the relationship between IDI1/SREBP2 axis and AIC.

### 4.5 Quantitative real-time (qRT) PCR

The RNA extraction kit was used to extract RNA from hepatic tissues. RNA concentrations were determined by Micro-Volume Measurement Platforms (BioDrop, USA) and uniformly diluted to 125 ng/μL. Completing DNA removal and RNA reverse transcription, respectively, with the RNA reverse transcription kit. The reverse transcription conditions is 37°C for 15 min and 85°C for 5 s. Next, amplification was performed. The amplification system includes 2 μL cDNA, 5 μL 2 × TB green, 0.4 μL forward primer (10 μm), 0.4 μL reverse primer (10 μm), and 2.2 μL ddH_2_O. The amplification condition is 95°C for 3 min, 40 cycles of 95°C for 12 s, and 62°C for 40 s. Each sample has three multiple holes. Its mean value was used for analysis. Livak (2^−ΔΔCT^) method was used to calculate RNA relative expression (Livak and Schmittgen, 2001). Primer sequences were designed and synthesized by Sangon Biotech (Shanghai) Co., Ltd. (Table 2).

**TABLE 2:** Sequencing information of hub genes.

Gene	Gene id	Primers (5′⟶3′)
IDI1	89784	F: GCT GCC GAG GTT GAA GTA C
		R: GCT GGC ATT GAT TTC AGG CAT TGT G
SREBP2	300095	F: AGC AAC AAC AGC AGT GGC AGA G
		R: GGT GGA TGA GGG AGA GAA GGT AGA C
LDLr	300438	F: CTT GTC CAT CTT CCT CCC CAT TGC
		R: ATC TCG TCC TCC GTG GTC TTC TG
HMGCR	25675	F: GAC CAA CCT TCT ACC TCA GCA AGC
		R: GGA CAA CTC ACC AGC CAT CAC AG
HMGCS1	29637	F: CGG TTC CCT TGC TTC TGT TCT GG
		R: CCT GGT GTG GCA TCT TGT GTG AC
LCN2	170496	F: AGC GAA TGC GGT CCA GAA AGA AAG
		R: CGA GGA TGG AAG TGA CGT TGT AGC
IL-1β	24494	F: CCC TGA ACT CAA CTG TGA AAT AGC A
		R: CCC AAG TCA AGG GCT TGG AA
TNF-α	24835	F: ATA CAC TGG CCC GAG GCA AC
		R: CCA CAT CTC GGA TCA TGC TTT C
Actin	81822	F:TGT TGC CCT AGA CTT CGA GCA
		R: CCA TAC CCA GGA AGG AAG GCT

### 4.6 Statistical analysis

Data were analyzed in IBM SPSS Statistics 25. Data were expressed as mean ± SD. One-way analysis of variance (ANOVA) was used to determine if there was a difference in results. A post-ANOVA Tukey (HSD) multiple mean comparison test was used to assess the specificity of the differences. *p*-value < 0.05 suggest the difference is significant.

## 5 Results

### 5.1 Results of HPLC-MS/MS

**FIGURE 2:**
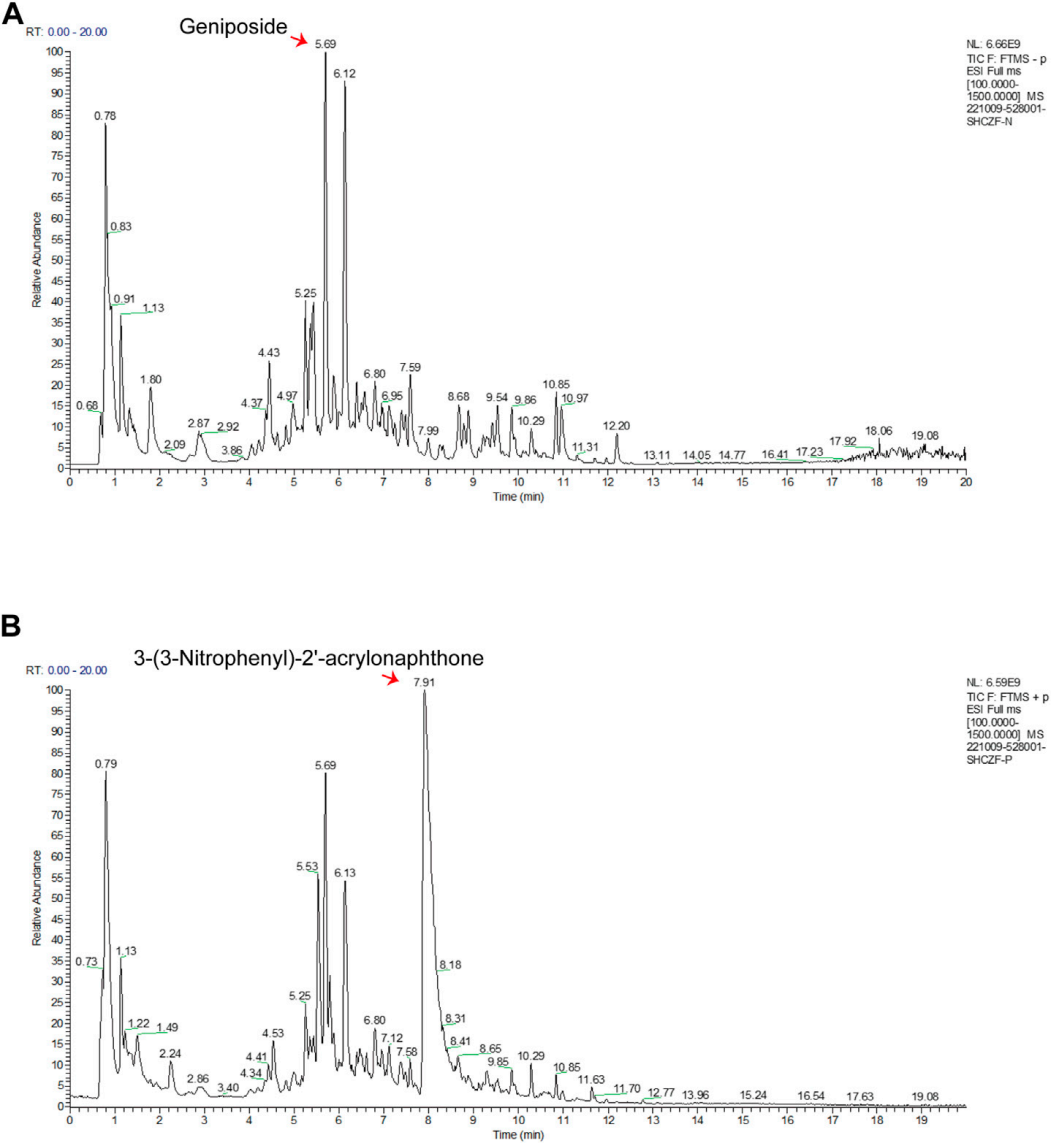
The results of HPLC-MS/MS. **(A)** Total ion current (TIC) chromatograms in negative mode, **(B)** TIC chromatograms in positive mode.

In the negative and positive modes, 240 and 207 bioactive substances in SHCZF were identified, respectively. Among them, geniposide and 3-(3-Nitrophenyl)-2′-acrylonaphthone were the most concentrated bioactive ingredients obtained in negative and positive ion modes (Figure 2). The relative concentration of geniposide was as high as 7114.3 μg/g, while that of 3-(3-Nitrophenyl)-2′-acrylonaphthone was 3460.4 μg/g. Geniposide, the bioactive ingredient of *Gardenia jasminoides Ellis.* (Zhi Zi), showed the highest concentration in SHCZF, which suggests that gardenia or Zhi Zi may be the critical component in SHCZF to develop its efficacy.

In addition, we further summarized the active ingredients with the highest concentration (top 10) in positive and negative ion modes. Table 3 shows that in addition to geniposide, the concentrations of rheic acid (1271.2 μg/g), the primary bioactive ingredient of *Rheum officinale Baill* (Da Huang) was equally high. The above results suggest that SHCZF may acquire tits he unique advantages in treating diseases through multiple targets and mechanisms because of the simultaneous cross-action of multiple herbs.

**TABLE 3:** Top 10 bioactive ingredients with the highest relative concentrations in SHCZF.

Bioacitve ingredient	Molecular	Class	Concentration (μg/g)
Geniposide	C_17_H_24_O_10_	Prenol lipids	7114.3
(3R,5R)-1,3,5-Trihydroxy-4-{[(2E)-3-(4-hydroxy-3-methoxyphenyl)-2-propenoyl]oxy}cyclohexanecarboxylic acid	C_17_H_20_O_9_	Organooxygen compounds	5782.4
3-(3-Nitrophenyl)-2′-acrylonaphthone	C_19_H_13_NO_3_	Linear 1,3-diarylpropanoids	3460.4
Gallic acid	C_7_H_6_O_5_	Benzene and substituted derivatives	2924.1
Methyl chlorogenate	C_17_H_20_O_9_	Organooxygen compounds	2510.9
Catechin	C_15_H_14_O_6_	Flavonoids	1887.4
N-[4-(Acetylsulfamoyl)phenyl]-2-phenyl-2-(phenylsulfanyl)acetamide	C_22_H_20_N_2_O_4_S_2_	Benzene and substituted derivatives	1672.7
6-O-[(2E)-3-Phenyl-2-propenoyl]-1-O-(3,4,5-trihydroxybenzoyl)-β-D-glucopyranose	C_22_H_22_O_11_	Tannins	1437.5
Rheic acid	C_15_H_8_O_6_	Anthracenes	1271.2
Sinapoylglucose	C_17_H_22_O_10_	Cinnamic acids and derivatives	1136.3

### 5.2 Results of bioinformatics analysis

**FIGURE 3:**
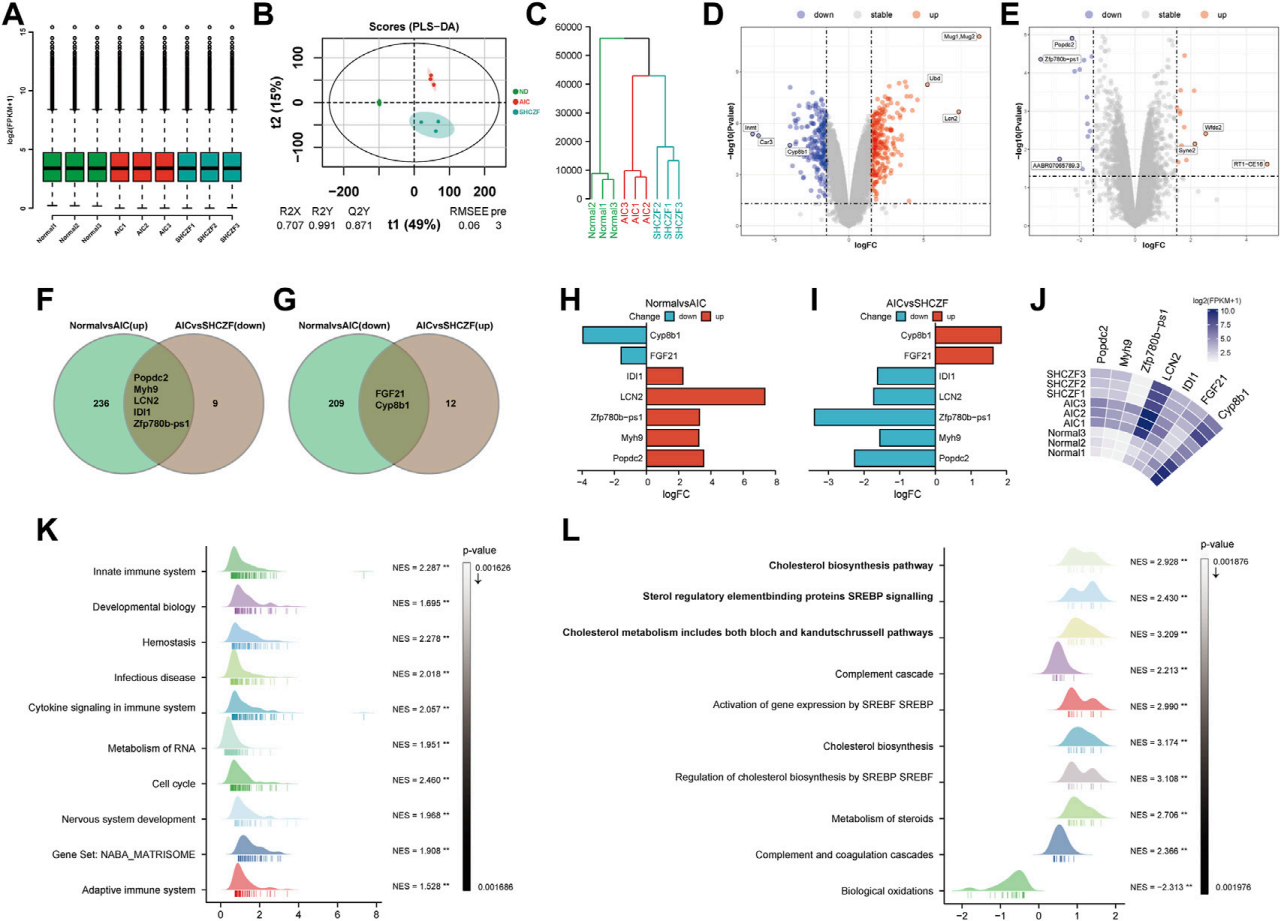
The results of bioinformatics analysis. **(A)** Boxplot, **(B)** PLS-DA plot, **(C)** Dendrogram, **(D,E)** Volcano plots, **(F,G)** Veen plots, **(H–J)** Crucial gene expression analysis plots, **(K,L)** GSEA analysis results.

Standardization analysis aims to remove biases in sequencing data caused by technology and their possible impact on subsequent analysis. Figure 3A suggests that the sequencing data used for analysis have been normalized. Figures 3B, C indicate that the expression levels in the normal, AIC, and SHCZF rats has a distribution difference, suggesting that the intervention of ANTI and SHCZF significantly affects the gene expression of rats.

The results of DEGs screening showed that, 241 genes were upregulated, and 211 genes were downregulated in AIC rats compared with normal rats. Mug1, LCN2, Ubd/Inmt, Car3, and Cyp8b1 were the top 3 genes with the most significant up/downregulation of expression (Figure 3D). Compared with AIC rats, SHCZF intervention resulted in upregulation of 14 genes and downregulation of 14 genes. RT1-CE16, syne2, Wfdc2, Zfp780b-ps1, AABR07065789.3, and Popdc2 were the top 3 genes with the most significant up/downregulation of expression, respectively (Figure 3E). A total of 7 genes were screened from the above genes by taking intersections, including Popdc2, Myh9, Zfp780b-ps1, LCN2, IDI1, FGF21, and Cyp8b1 (Figures 3F, G). The expression levels of Popdc2, Myh9, Zfp780b-ps1, Lcn2, and Idi1 were increased in the ANTI-induced AIC rats (Normal vs. AIC) and decreased after SHCZF treatment (AIC vs. SHCZF). The expression of FGF21 and Cyp8b1 was downregulated after ANTI stimulation (Normal vs. AIC) and upregulated after SHCZF treatment (Figures 3H–J). Based on the expression changes of the above genes in the normal, AIC, and SHCZF rats (elevated or decreased in the disease model and reversed after SHCZF treatment), we speculated that the above genes might be AIC-related disease genes and therapeutic target genes of SHCZF.

GSEA results suggested that the mechanism of ANTI-induced AIC in rats may be immune-related (Figure 3K). The process of cholestasis induced by toxic ANTI in rats is an invasion of a foreign substance, which will activate the immune system of rats. SHCZF treats the AIC of rats by improving the metabolism of sterols, especially cholesterol (Figure 3L). GSEA enrichment results (AIC vs. SHCZF) showed that IIDI1, Sqle, Hmgcs1, Cyp51, Fdps, and Fdft1 simultaneously exist in the top 3 significant pathways (*p*-value minimum), suggesting that these genes may be the core genes in SHCZF-related cholesterol regulatory pathways (Table 4).

**TABLE 4:** GSEA crucial pathways information Table (AIC vs. SHCZF).

ID	*p*-value	Core genes
Cholesterol biosynthesis pathway	0.001876	IID1, Sqle, Hmgcs1, Cyp51, Fdps, Fdft1, Msmo1, Mvd, Sc5d, Mvk, Pmvk
Sterol regulatory element binding proteins SREBP signaling	0.001883	IDI1, Sqle, Hmgcs1, Cyp51, Fdps, Fdft1, Insig1, Mvd, Fasn
Cholesterol metabolism includes both bloch and kandutschrussell pathways	0.001894	IDI1, Sqle, Hmgcs1, Cyp51, Fdps, Fdft1, Hsd17b7, Msmo1, Mvd, Ebp, Sc5d, Dhcr24, Fasn, Mvk, Pmvk

Among the above genes, only IDI1 is a target gene of both ANTI, SHCZF and the core gene in cholesterol metabolism-related pathways. The change in the expression of IDI1 may be the key to SHCZF treating AIC rats and may be related to cholesterol metabolism.

### 5.3 Formulation of a hypothesis

IHC is a pathophysiological process caused by bile secretion and excretion disorders, characterized by excessive accumulation of bile components such as cholesterol and bilirubin in intrahepatic bile ducts, causing damage to the liver.

IDI1 is an essential metabolic gene related to cholesterol synthesis and transport (Yang et al., 2022). IDI1 can regulate cholesterol synthesis and transport through SREBP2 (Tremblay et al., 2011; Zhang et al., 2012; Zhang et al., 2018). The upregulation of LDLr caused by SREBP2 activation promotes the uptake of free cholesterol, which is an essential reason for cholesterol accumulation (Van Rooyen et al., 2011). SREBP2 can also directly regulate the rate of cholesterol synthesis by regulating the expression of the rate-limiting enzymes HMGCR (Poornima et al., 2022) and HMGCS1 (Vock et al., 2008; Madison, 2016). Excessive accumulation of cholesterol in the liver will stimulate the expression of LCN2, causing inflammation and subsequent hepatic damage (Ye et al., 2016; Currò et al., 2020).

**FIGURE 4:**
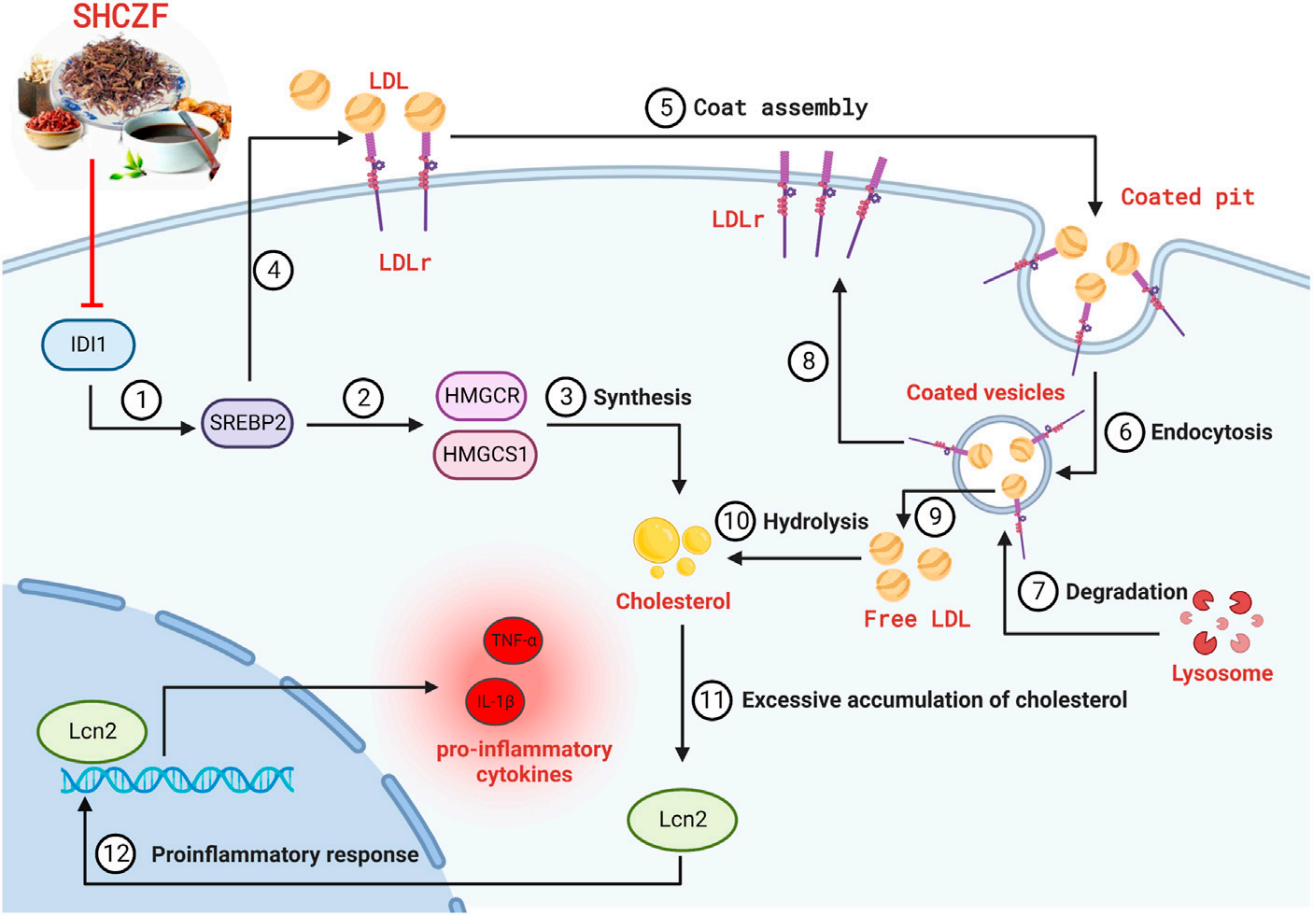
The hypothesis of SHCZF in treating AIC rats. ②, ③The mechanism of cholesterol synthesis, ④–⑨The mechanism of cholesterol uptake: Endocytosis mediated by the LDLr on the cell surface can absorb cholesterol-rich LDL from the blood, called the LDLr pathway. LDLr can combine with free LDL in plasma to form an LDL-LDLr complex, which appears in the coated pit, and forms coated vesicles to enter the cell. The outer skin of coated vesicles is then depolymerized, causing the separation of LDL and LDLr. LDLr returns to the cellular membrane for the next cycle, while LDL is degraded by lysosomes and hydrolyzed into free cholesterol.

Therefore, we propose the following hypothesis based on bioinformatics and literature analysis results. SHCZF reduces LDL-c uptake by regulating IDI1/SREBP2/LDLR pathway. SHCZF can also lower cholesterol synthesis in the liver by regulating IDI1/SREBP2/HMGCR (HMGCS1) pathway (Figure 4).

### 5.4 Results of experiments

#### 5.4.1 SHCZF protects rats from ANTI-induced AIC

**FIGURE 5:**
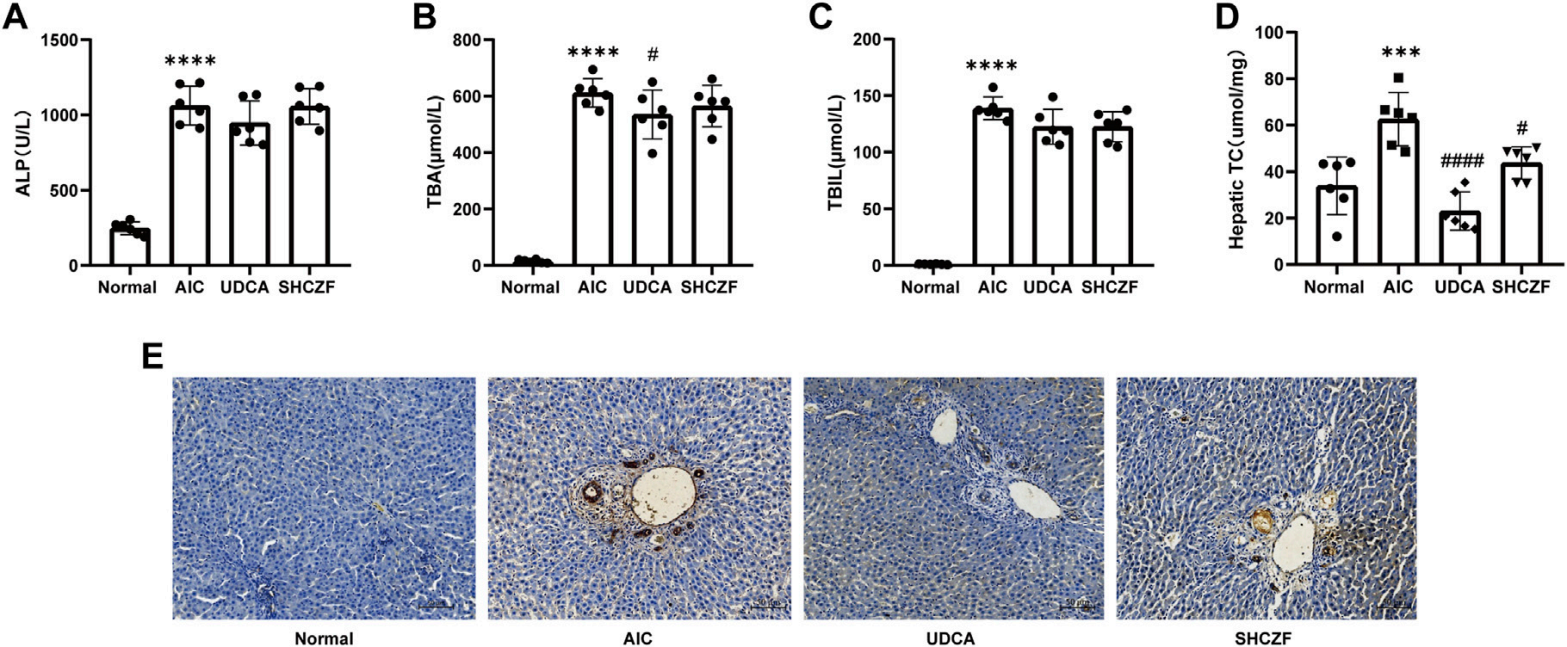
The results of cholestasis levels in rats. **(A)** ALP level, **(B)** TBA level, **(C)** TBIL level, **(D)** Hepatic cholesterol content level, **(E)** CK19 immunohistochemistry (400x).

Figures 5A–C showe that the cholestasis-related indicators ALP, TBA, and TBIL were significantly higher in the serum of AIC rats than in the serum of normal rats (*p* < 0.05). After SHCZF and UDCA administration, the concentration of these indicators in the serum of rats was reduced. Intrahepatic cholesterol levels were significantly higher in AIC rats than that in normal rats (*p* < 0.05) and decreased after UDCA and SHCZF treatment (*p* < 0.05). SHCZF restored hepatic cholesterol to levels similar to those in normal rats, while UDCA restored cholesterol to levels below those in normal rats (Figure 5D). The common pathological manifestations of IHC include cholestasis starting from hepatocytes in the third region of the hepatic lobules, manifesting as feathery degeneration of hepatocytes with dilated bile ducts (Nakanuma et al., 2012). Cytokeratin 19 (CK19) is a bile duct type cytokeratin. Immunohistochemistry of CK19 can effectively reflect bile duct dilatation in the liver. Figure 5E shows increased bile duct dilatation in AIC rats compared to normal rats and significant improvement after SHCZF and UDCA treatment.

The above results suggest that both SHFZF and UDCA can improve hepatic cholestasis in rats. The efficacy of UDCA is better than that of SHCZF.

#### 5.4.2 SHCZF protects rats from hepatic injury

**FIGURE 6:**
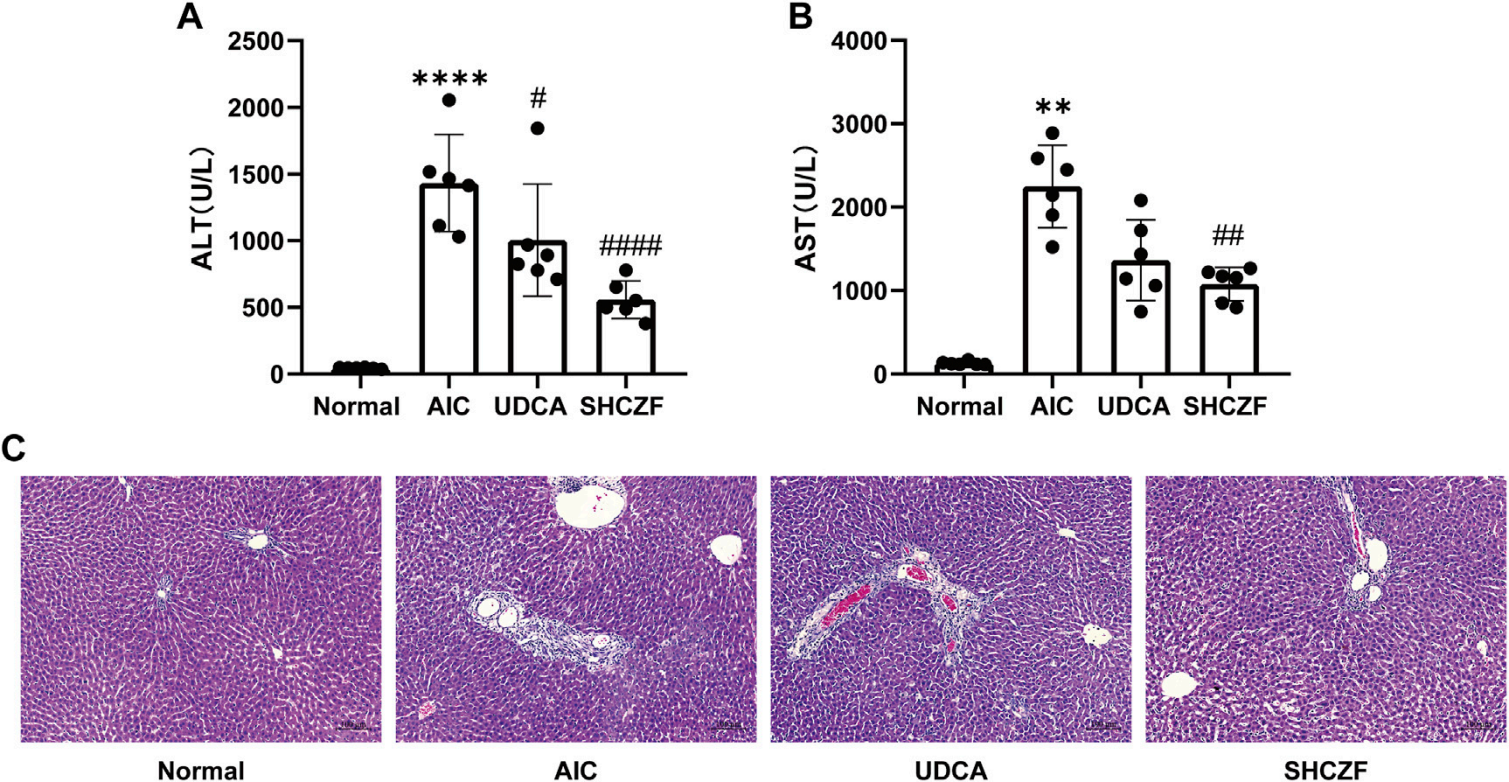
The results of hepatic injury in rats. **(A)** ALT level, **(B)** AST level, **(C)** HE staining (200x).

Figures 6A, B show that the hepatic injury-related indicators ALT and AST were significantly upregulated in AIC rats compared with normal rats (*p* < 0.05). After the administration of SHCZF and UDCA, ALT and AST were significantly reduced (*p* < 0.05). Figure 6C shows that AIC rats had significant inflammatory cell infiltration in the portal area, while SHCZF and UDCA effectively improved inflammation in the rat liver.

The above results suggest that both SHFZF and UDCA can improve hepatic inflammation and injury in rats. The efficacy of SHCZF is better than that of UDCA.

#### 5.4.3 Cholestasis precedes hepatic injury

When the liver is damaged, the transaminase in liver cells will be released into the blood, causing an increase in serum ALT and AST levels (Pol et al., 1991; Prati et al., 2002). The production of ALP can be induced when bile is not efficiently excreted and the internal pressure of bile capillaries is increased. Subsequently, bile acid dissolves ALP from the lipid membrane, increasing serum ALP levels (Siddique and Kowdley, 2012). Therefore, serum ALT and AST levels always reflect the degree of hepatic injury, while ALP demonstrates the level of cholestasis.

**FIGURE 7:**
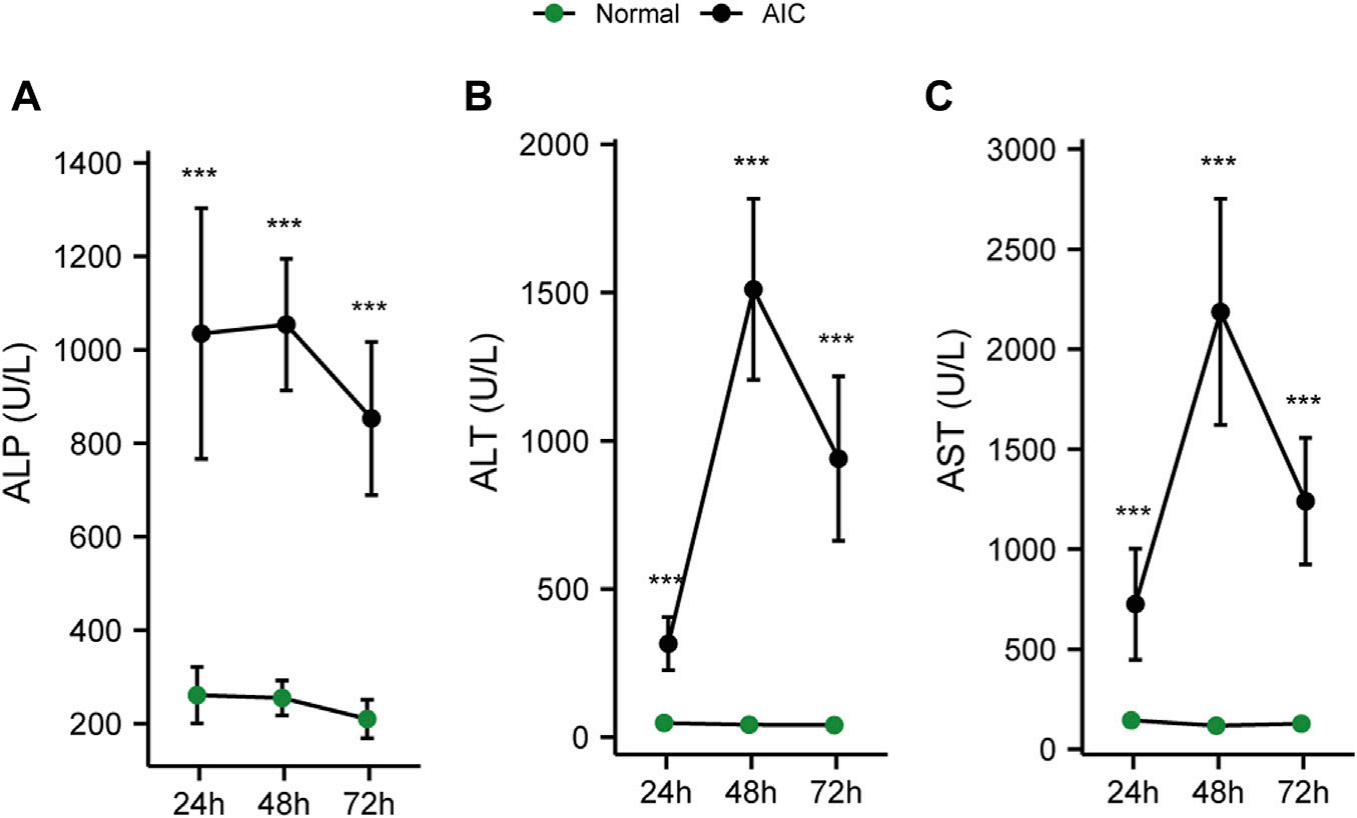
Dynamic chart of serum cholesterol and hepatic injury-related indicators in rats. **(A)** ALP dynamic change, **(B)** ALT dynamic change, **(C)** AST dynamic change.

To clarify the sequence of cholestasis and hepatic injury, we constructed 24, 48, 72 h AIC rat models and corresponding normal controls. Then, we detected the dynamic changes in ALT, AST, and ALP in rat serum. The results showed that compared with normal rats, the level of serum ALP in AIC rats increased significantly at 24 h (Figure 7A). In comparison, ALT and AST levels increased dramatically after 24 h, reaching the peak at 48 h (Figures 7B, C). The ALP level was significantly higher in AIC rats than that in normal rats at 24 h, while ALT and AST did not increase considerably, suggesting that cholestasis may precede hepatic injury in rats.

#### 5.4.4 SHCZF inhibits IDI1/SREBP2/LDLr and IDI1/SREBP2/HMGCR/HMGCS1

IDI1 and SREBP2 are the crucial target genes of SHCZF for the treatment of cholestasis, as clarified by bioinformatics and literature analysis. Downstream LDLr (cholesterol uptake) and HMGCR/HMGCS1 (cholesterol synthesis) are controlled by SREBP2 for the critical genes for regulating cholesterol metabolism (Haskins et al., 2015; Che et al., 2020; Lebeau et al., 2022).

**FIGURE 8:**
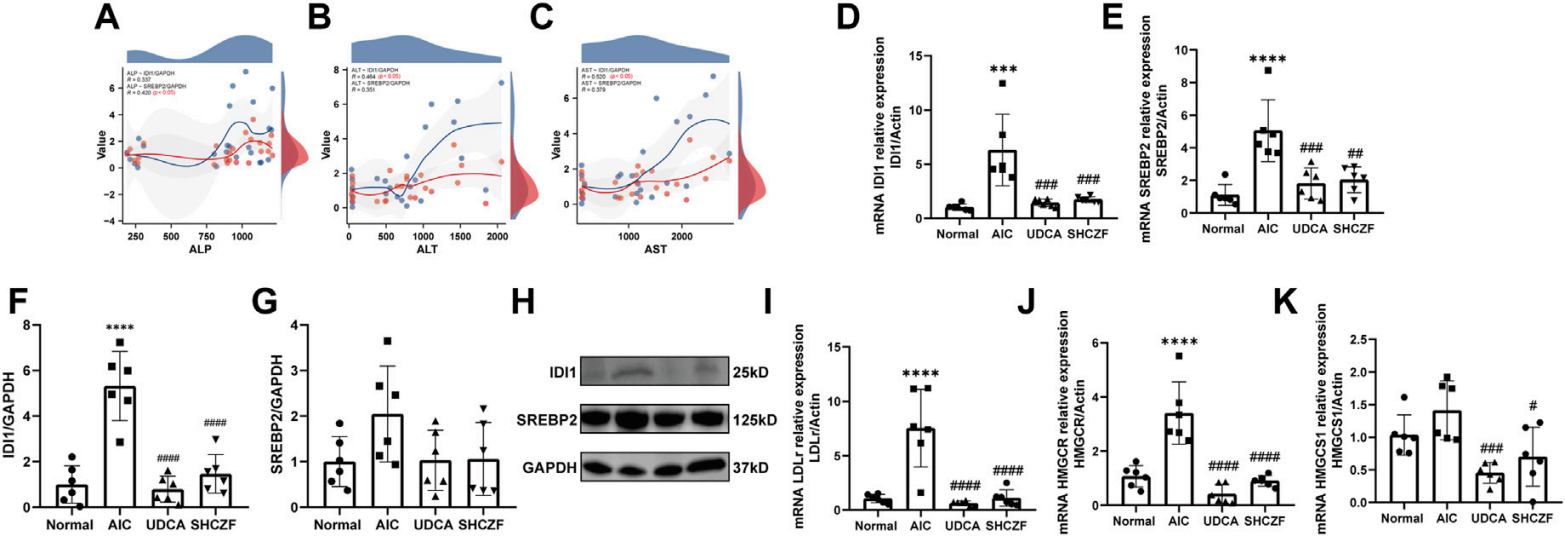
Expression of SHCZF target genes and cholesterol metabolism-related genes. **(A)** Correlation between IDI1, SREBP2, and ALP, **(B)** Correlation between IDI1, SREBP2, and ALT, **(C)** Correlation between IDI1, SREBP2, and AST, **(D)** mRNA expression of IDI1, **(E)** mRNA expression of SREBP2, **(F)** protein expression of IDI1, **(G)** protein expression of SREBP2, **(H)** WB bands of IDI1, SREBP2, and GAPDH, **(I)** mRNA expression of LDLr, **(J)** mRNA expression of HMGCR, **(K)** mRNA expression of HMGCS1.

The correlation coefficient (R) > 0 indicates a positive correlation between the two variables; Conversely, it is a negative correlation. The absolute value of correlation coefficient reflects the degree of correlation: 0–0.3 represents weak or uncorrelated; 0.3–0.5 represents weak correlation; 0.5–0.8 represents moderate correlation; 0.8–1 stands for strong correlation. The results of correlation analysis indicated that the expression of IDI1 and SREBP2 were at least weakly correlated with ALP, AST, and ALT levels (Figures 8A–C).

Figures 8D–H show that both the mRNA and protein expression of IDI1 and SREBP2 were increased after ANTI induction (*p* < 0.05), while intervention with SHCZF and UDCA significantly decreased their expression (*p* < 0.05). The expression of LDLr, HMGCR, and HMGCS1 was substantially higher in the liver tissues of AIC rats compared with normal rats (*p* < 0.05), while SHCZF and UDCA treatment significantly reduced the expression of these genes (*p* < 0.05) (Figures 8I–K). The expression change in the IDI1/SREBP2 pathway and downstream cholesterol uptake and synthesis-related hub genes met expectations. In addition, the efficacy of SHCZF in improving cholesterol metabolism was similar to that of UDCA.

#### 5.4.5 SHCZF reduces the release of inflammatory cytokines

Excessive accumulation of cholesterol activates the expression of LCN2. LCN2 substantially activates proinflammatory cytokines (TNF-α and IL-1β), causing hepatic inflammation (Weerachayaphorn et al., 2014; Ye et al., 2016).

**FIGURE 9:**
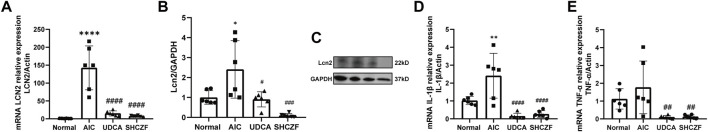
Expression of LCN2 and pro-inflammatory cytokines. **(A)** mRNA expression of LCN2, **(B)** protein expression of LCN2, **(C)** WB bands of Lcn2 and GAPDH, **(D)** mRNA expression of TNF-α, **(E)** mRNA expression of IL-1β.

Both the mRNA and protein expression levels of LCN2 increased after ANTI induction (*p* < 0.05), whereas intervention with SHCZF and UDCA decreased LCN2 expression (*p* < 0.05) (Figures 9A–C). Figures 9D, E shows that TNF-α and IL-1β were upregulated in AIC rats compared with normal rats (*p* < 0.05). SHCZF and UDCA treatment significantly reduced the expression levels of these proinflammatory cytokines (*p* < 0.05). The expression changes of LCN2 and downstream proinflammatory cytokines meets expectations. In addition, the efficacy of SHCZF in improving inflammation was better than that of UDCA.

## 6 Discussion

IHC is gaining increasing attention due to its high morbidity and severe outcome. However, until now, the drugs available for treating IHC have been quite limited. UDCA is one of the few prescription drugs approved by the U.S. Food and Drug Administration (FDA) for clinical use in treating IHC (Beuers et al., 1998). However, the therapeutic index (TI) of UDCA is narrow. Inappropriate therapeutic doses may cause “unintended” adverse reactions, such as hepatitis, ascites, severe diarrhea, and immunosuppression, etc. (Chapman, 2010; Sinakos et al., 2010). *In vivo*, UDCA can even transform into toxic lithocholic acid (LCA), which may induce death from liver failure in patients with impaired sulfation (one of the major conjugation pathways in the body with an essential role in human metabolism (Alnouti, 2009; Kotb, 2012). Finding reliable drugs for the treatment of cholestasis is critical.

In China, traditional Chinese medicine has been used to treat diseases for thousands of years and shows great value for rediscovery. Among the medicines, SHZCF, originating from *Jin Gui Yao Lue*, is a traditional Chinese formula used for treating cholestasis in the clinic. In a previous study, we have clarified the mechanism by which SHCZF improves cholestasis through mitochondrial oxidative stress (Liu et al., 2022b). We also screened essential bioactive ingredients and possible target genes of SHCZF through network pharmacology (Liu et al., 2022a). However, no study has directly confirmed the effect of SHCZF on cholesterol metabolism-related pathways. In addition, the relationship between cholestasis and liver inflammation and the improvement effect of SHCZF is also unclear.

In this study, through transcriptome sequencing data and bioinformatics analysis, we preliminarily determined that IDI1/SREBP2, a cholesterol metabolism pathway, may be the therapeutic target of SHCZF. The results of CK19 immunohistochemistry, TC, and evaluation of cholestasis-related indicators showed that the intervention of SHCZF and UDCA effectively improved the bile duct dilatation and cholestasis in the rat liver. The effect of UDCA on improving cholesterol was significantly higher than that of SHCZF, which could reduce cholesterol to a level lower than that of normal rats. However, a moderate amount of cholesterol is beneficial. Studies have found that cholesterol can induce oxidative stress, mitochondrial damage, and the death of hepatic stellate cells to reduce the degree of fibrosis (Rauchbach et al., 2022). Moderate cholesterol can also activate Nrf-2 and HIF-1α and promote hepatocyte proliferation and liver regeneration, improving hepatic fibrosis (Kaminsky-Kolesnikov et al., 2020). SHCZF, which restored the cholesterol level in the liver of rats to approximately that in normal rats, may be more suitable for the treatment of cholestasis. The dynamic rat model confirmed that continuous cholestasis further led to hepatic inflammation and subsequent damage, reflecting the importance of timely treatment of cholestasis. Assessment of hepatic injury indicators (transaminase) and pro-inflammatory cytokines showed that both SHCZF and UDCA improved hepatic inflammation and injury, but the improvement effect of SHCZF was better. Based on the analysis of the experimental results, we confirmed that SHCZF can indeed improve cholestasis and subsequent hepatic inflammation and injury by regulating IDI1/SREBP2 pathway. Simultaneously, through the comparison of curative effects, we believe that SHCZF may be more effective than the positive drug UDCA. However, the above analysis results only come from rat experiments, lacking the support of clinical research. As a complex drug composed of various traditional Chinese herbs, SHCZF quality control is also an unavoidable problem.

## 7 Conclusion

In this study, we confirmed the efficacy of SHCZF improving ANTI-induced AIC in rats: 1) Cholesterol sludge and inflammatory infiltration in the rat liver were alleviated; 2) IDI1/SREBP2 expression was reduced, and cholesterol uptake (LDLr), synthesis (HMGCR/HMGCS1)-related gene expression was inhibited; 3) Excessive accumulation of cholesterol in the liver may further induce the expression of LCN2, causing hepatic inflammation and injury; 4) The expression of LCN2 was significantly reduced, and the levels of proinflammatory cytokines TNF-α and IL-1β were suppressed.

## Data Availability

The datasets presented in this study can be found in online repositories. The names of the repository/repositories and accession number(s) can be found below: NCBI BioProject https://www.ncbi.nlm.nih.gov/bioproject/, PRJNA906447.

## Supplementary material

Supplementary Table 1 UHPLC-MSMS results.

Supplementary Table 2 WB original data.

Supplementary Table 3 GSEA results (AIC vs SHCZF).

Supplementary Table 4 Biochemical index of disease model.

Supplementary Table 5 Biochemical index of dynamic model.

Supplementary Table 6 GSEA results (ND vs AIC).

Supplementary Table 7 PCR original data.

Supplementary Table 8 RNA control.

Supplementary Table 9 TC original data.

Supplementary Table 10 WB original bands.

## Abbreviations

AIC, Acute intrahepatic cholestasis; ANTI, Alpha-naphthylisothiocyanate; AAAALAC, Association for Assessment and Accreditation of Laboratory Animal Care; CLD, Cholestatic liver diseases; DHXSD, Da-Huang-Xiao-Shi decoction; DEGs, differential expressed genes; GGT, Gamma-glutamyl transaminase; HPLC, High-performance liquid chromatography; IACUC, Institutional Animal Care and Use Committee; ICP, Intrahepatic cholestasis of pregnancy; IHC, Intrahepatic Cholestasis; IDI1, Isopentenyl-Diphosphate Delta Isomerase 1; IL-1β, Interleukin 1 Beta; LCA, Lithocholic acid; LCN2, Lipocalin 2; LDLr, Low-Density Lipoprotein Receptor; PLS-DA, Partial least squares discrimination analysis; PFIC, Progressive familial intrahepatic cholestasis; qRT-PCR, Quantitative real-time PCR; SD, Sprague-Dawley; SHCZF, San-Huang-Cai-Zhu formula; SREBP2, Sterol Regulatory Element-Binding Protein 2; TCM, Traditional Chinese medicine; TI, Therapeutic index; TNF-α, Tumor Necrosis Factor Alpha; UDCA, Ursodeoxycholic Acid; HPLC-MS/MS, High-Performance Liquid Chromatography with Tandem Mass Spectrometry; WB, Western blotting.
