# Supplementary figures and images for: The IDI1/SREBP2 axis drives intrahepatic cholestasis and is a treatment target of San-Huang-Cai-Zhu formula identified by sequencing and experiments
Source: Front Pharmacol. 2023 Feb 8;14:1093934. doi: 10.3389/fphar.2023.1093934 (PMC9944032; doi:10.3389/fphar.2023.1093934)

**GAPDH**


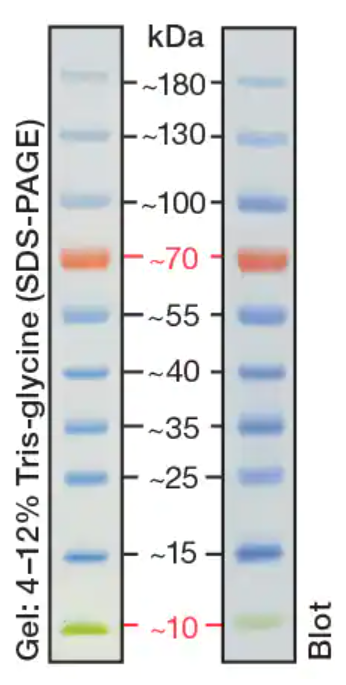


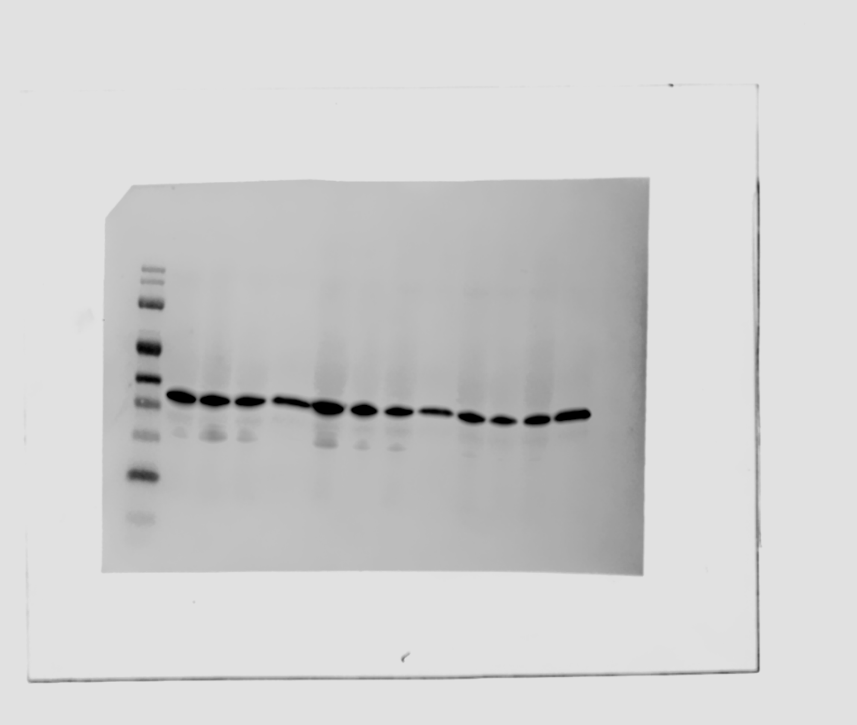


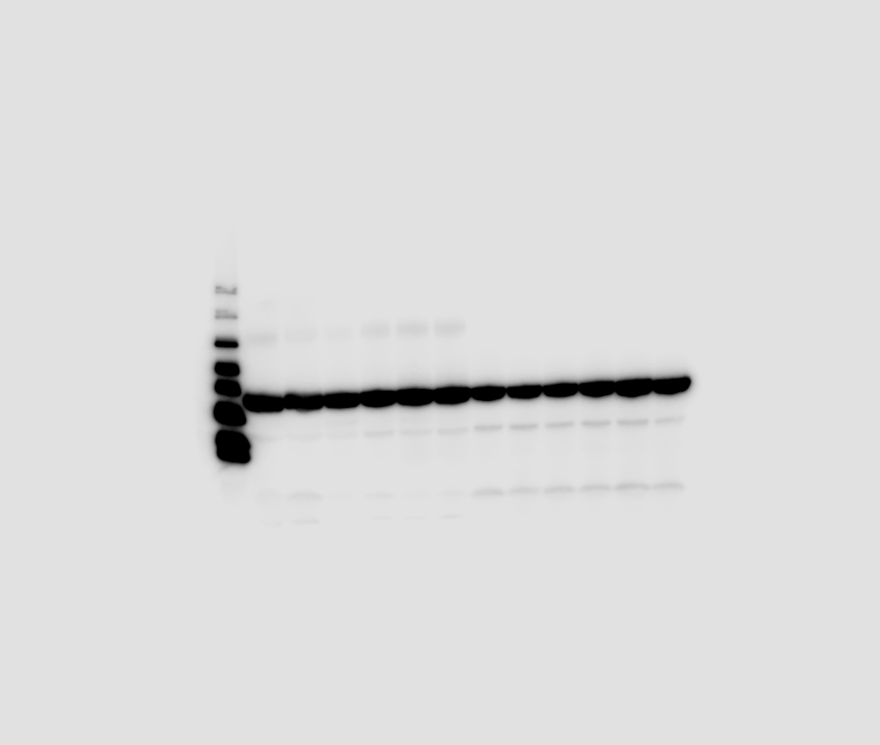


**IDI1**


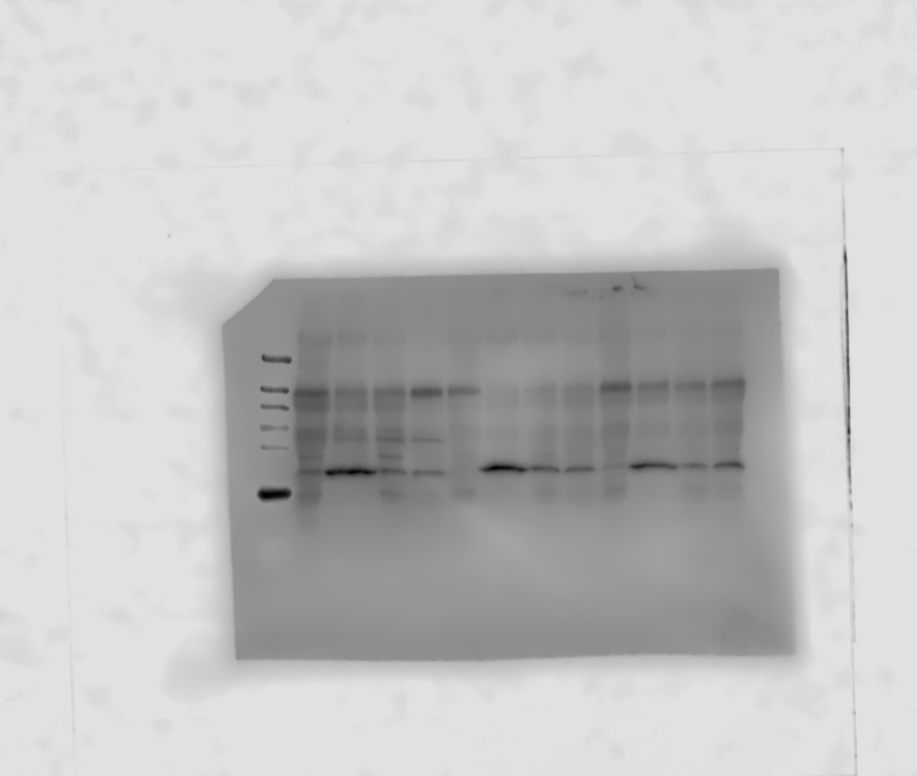

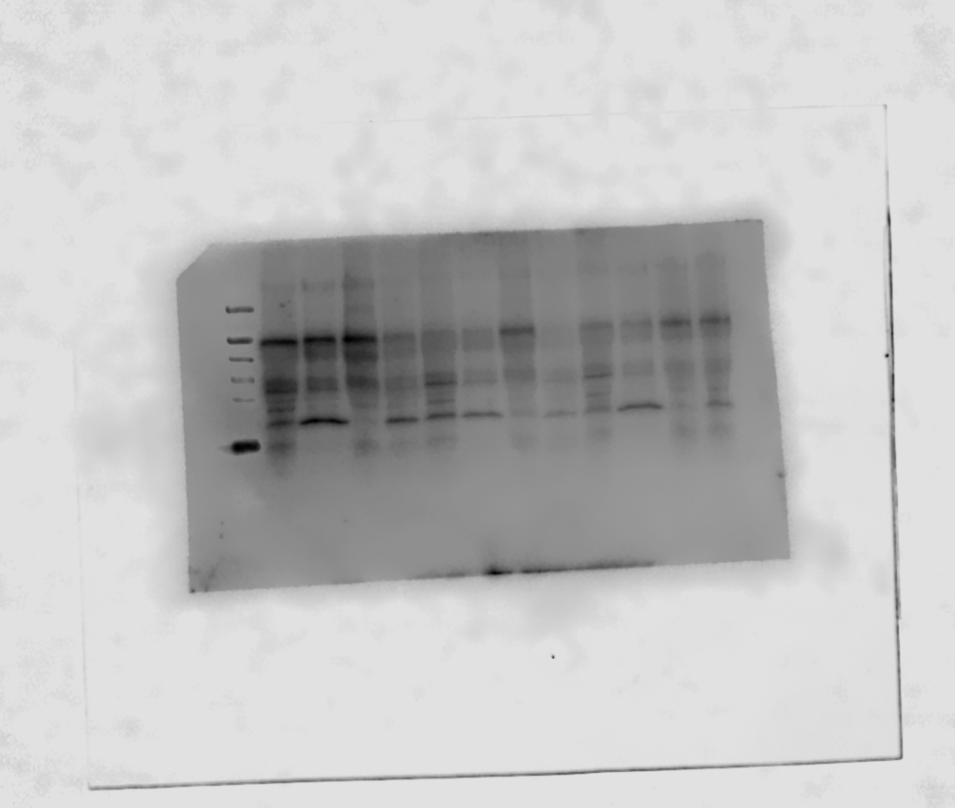

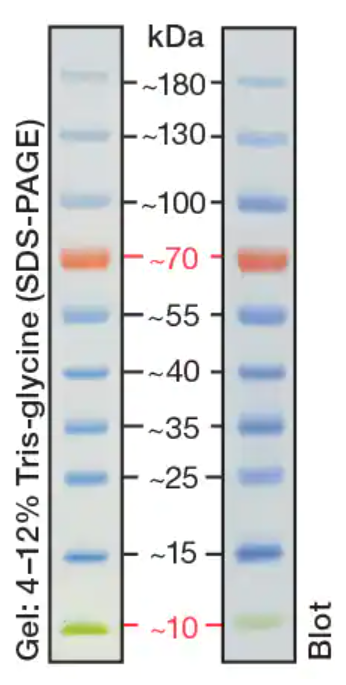


**LCN2**


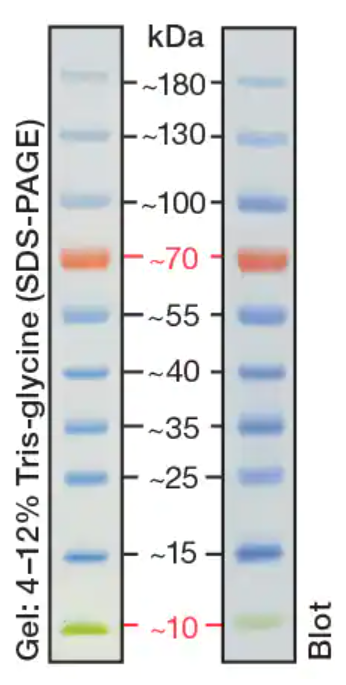


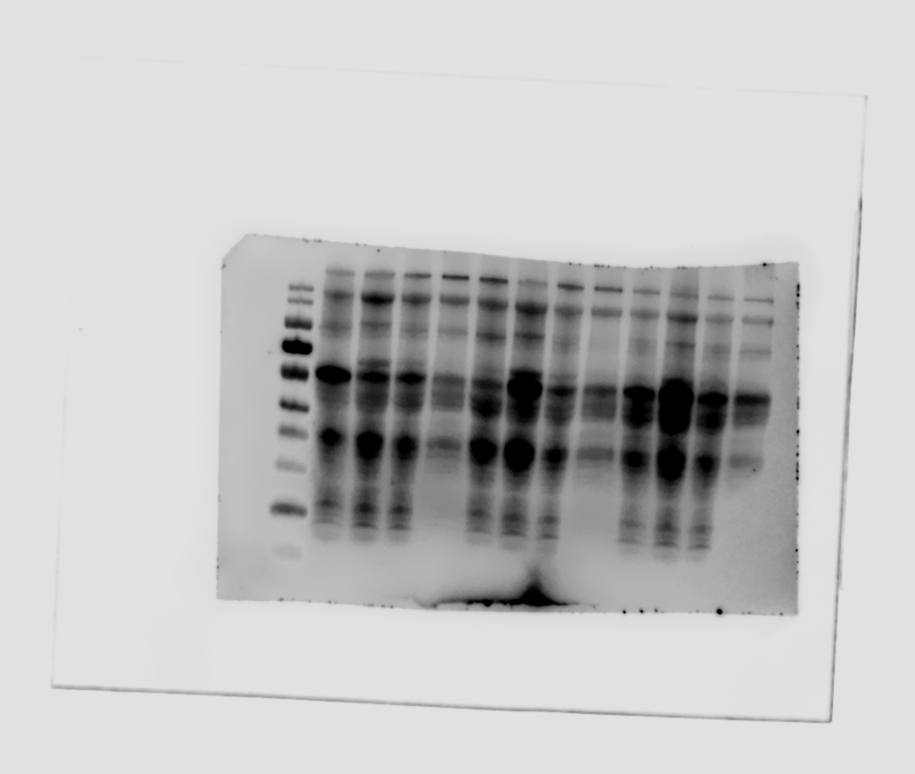


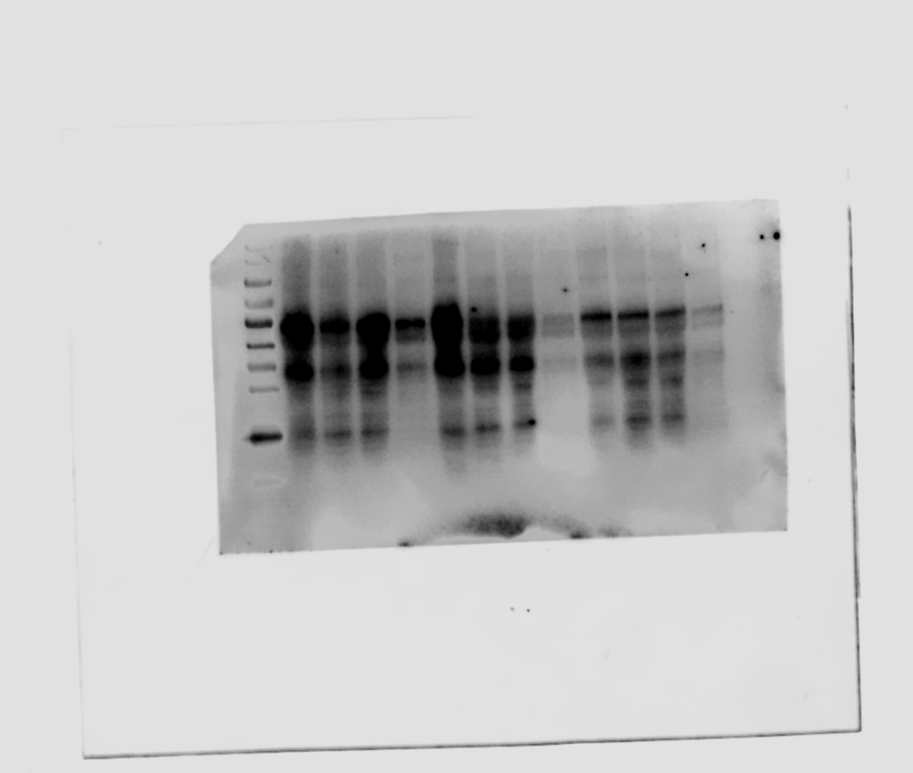


**SREBP2**


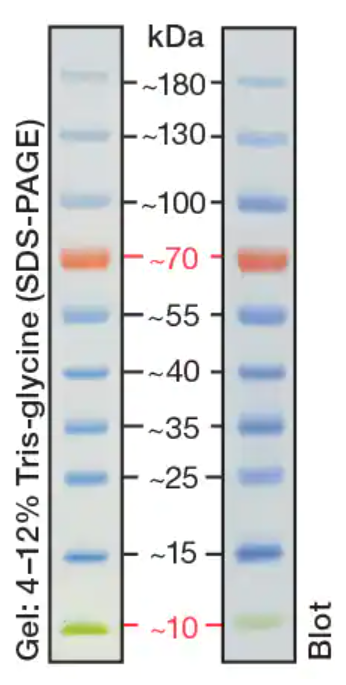


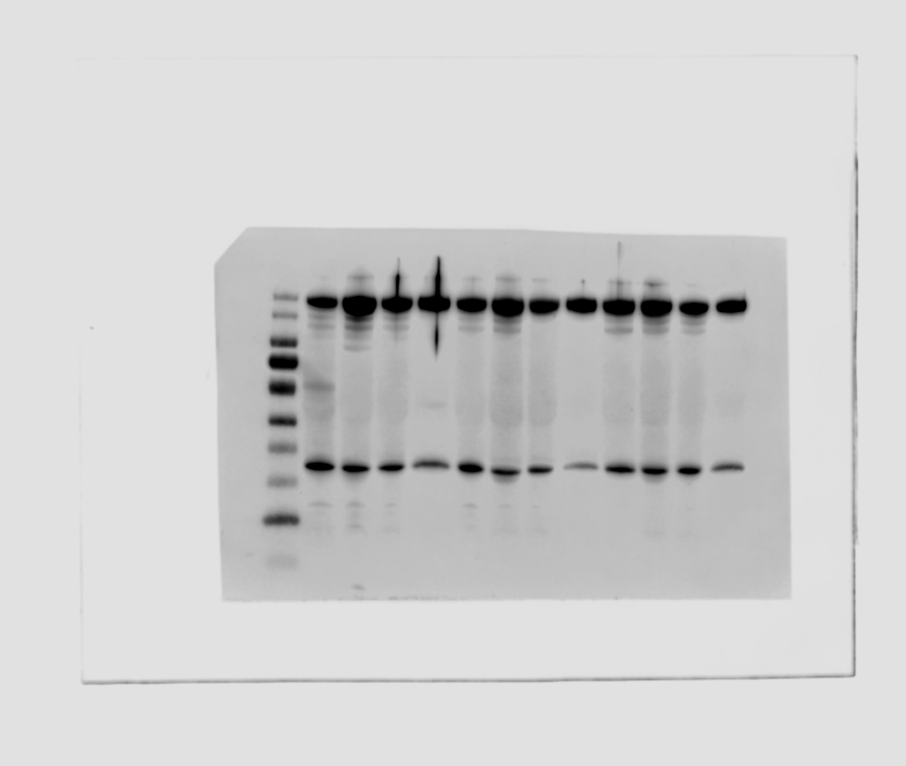


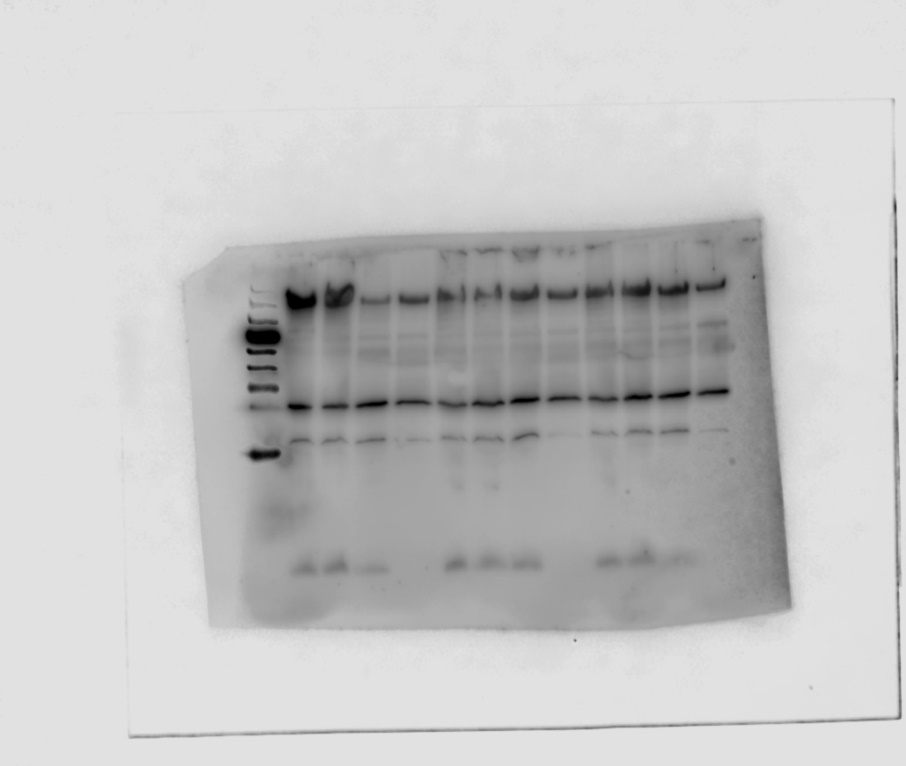

Supplement: Supplementary file 10 [file Table10.DOCX]
